# Evidence for chemokine synergy during neutrophil migration in ARDS

**DOI:** 10.1136/thoraxjnl-2016-208597

**Published:** 2016-08-05

**Authors:** Andrew E Williams, Ricardo J José, Paul F Mercer, David Brealey, Dhruv Parekh, David R Thickett, Cecelia O'Kane, Danny F McAuley, Rachel C Chambers

**Affiliations:** 1 Division of Medicine, Centre for Inflammation and Tissue Repair, UCL Respiratory, Rayne Institute, University College London (UCL), London, UK; 2 Bloomsbury Institute of Intensive Care Medicine, University College Hospital, London, UK; 3 Institute of Inflammation and Aging, University of Birmingham, Birmingham, UK; 4 Centre for Experimental Medicine, Wellcome-Wolfson Institute for Experimental Medicine, Queen's University of Belfast and Regional Intensive Care Unit, Royal Victoria Hospital, Belfast, UK

**Keywords:** Cytokine Biology, Innate Immunity, Neutrophil Biology, ARDS

## Abstract

**Background:**

Acute respiratory distress syndrome (ARDS) is a life-threatening condition characterised by pulmonary oedema, respiratory failure and severe inflammation. ARDS is further characterised by the recruitment of neutrophils into the lung interstitium and alveolar space.

**Objectives:**

The factors that regulate neutrophil infiltration into the inflamed lung and our understanding of the pathomechanisms in ARDS remain incomplete. This study aimed at determining the role of the chemokine (C-C motif) ligand (CCL)2 and CCL7 in ARDS.

**Methods:**

CCL2 and CCL7 protein levels were measured in bronchoalveolar lavage (BAL) fluid obtained from lipopolysaccharide(LPS)-challenged human volunteers and two separate cohorts of patients with ARDS. Neutrophil chemotaxis to ARDS BAL fluid was evaluated and the contribution of each was assessed and compared with chemokine (C-X-C motif) ligand 8 (CXCL8). Chemokine receptor expression on neutrophils from blood or BAL fluid of patients with ARDS was analysed by flow cytometry.

**Results:**

CCL2 and CCL7 were significantly elevated in BAL fluid recovered from LPS-challenged volunteers and patients with ARDS. BAL fluid from patients with ARDS was highly chemotactic for human neutrophils and neutralising either CCL2 or CCL7 attenuated the neutrophil chemotactic response. Moreover, CCL2 and CCL7 synergised with CXCL8 to promote neutrophil migration. Furthermore, neutrophils isolated from the blood or BAL fluid differentially regulated the cell surface expression of chemokine (C-X-C motif) receptor 1 and C-C chemokine receptor type 2 during ARDS.

**Conclusion:**

This study highlights important inflammatory chemokines involved in regulating neutrophil migration, which may have potential value as therapeutic targets for the treatment of ARDS.

**Key messages**

**What is the key question?**

- To what extent do the chemokine (C-C motif) ligand (CCL)2 and CCL7 contribute to the migratory activity of neutrophils during acute respiratory distress syndrome (ARDS)?

**What is the bottom line?**

- Both CCL2 and CCL7 contribute to neutrophil chemotaxis during ARDS by synergising with chemokine (C-X-C motif) ligand 8.

**Why read on?**

- Excessive neutrophil recruitment during ARDS is associated with disease severity and poor clinical outcome; therefore, a clearer understanding of these pathomechanisms is urgently needed.

## Introduction

Acute respiratory distress syndrome (ARDS) is a life-threatening condition that is still associated with a high rate of mortality despite recent improvements in mechanical ventilation strategies and supportive care. ARDS is characterised by lung oedema, presenting as diffuse bilateral lung opacities and hypoxaemia that does not arise from cardiac failure or fluid overload.^1^ ARDS can be classified as mild, moderate or severe, depending on the partial pressure of arterial oxygen to fraction of inspired oxygen ratios (PaO_2_/FIO_2_). ARDS remains a syndrome resulting from varied aetiologies and is associated with multiple pathologies, of which diffuse alveolar damage remains the most common pathognomonic feature.^2^

Disease onset is often rapid and progressive and is usually the result of either a direct insult to the lung, such as pneumonia, aspiration of gastric contents or pulmonary contusion or the indirect insults such as non-pulmonary sepsis, polytrauma or transfusion-associated acute lung injury. ARDS is further associated with acute inflammation^3^ and the rapid accumulation of neutrophils in the lung interstitium and alveolar space.^4^ In addition, neutrophil counts remain higher in the bronchoalveolar lavage (BAL) fluid of non-survivors with ARDS compared with survivors.^5^ The excessive accumulation of neutrophils in ARDS has therefore been directly implicated in disease pathogenesis and poor clinical outcome.^6^

The migration of neutrophils into inflamed lungs is mediated by multiple factors, of which cell adhesion molecules and chemokines are thought to be the most important.^7^ The chemokine (C-X-C motif) ligand 8 (CXCL8) (interleukin (IL)-8) is considered to be the archetypal neutrophil chemoattractant. Indeed, the levels of CXCL8 have been positively correlated with the number of neutrophils recovered from patients with ARDS^8^ and with disease severity.^9^ ^10^ However, several studies have reported that other chemokines may be elevated in ARDS.^11^ Furthermore, we have recently reported that neutrophil recruitment in the lung airspaces, in response to lipopolysaccharide(LPS)-induced lung inflammation, is mediated by the chemokine (C-C motif) ligand (CCL)2 and CCL7,^12^ while CCL7 regulates neutrophil recruitment in response to acute infection with the bacterium *Streptococcus pneumoniae*.^13^

In this report we aim to gain a better understanding of the potential roles of CCL2 and CCL7 in ARDS by focussing on their contribution to neutrophil recruitment. To this end we measured the levels of CCL2 and CCL7 in samples obtained from a human LPS-challenged model of acute lung injury and two separate cohorts of patients with ARDS. We investigated the contribution of CCL2 and CCL7 to the neutrophil chemotactic activity of human ARDS BAL fluid and assessed the chemotactic response of neutrophils to CCL2 and CCL7 in the context of the classical neutrophil chemokine CXCL8. Finally, we analysed the chemokine receptor expression on neutrophils derived from the blood and BAL fluid of patients with ARDS to determine if the receptor expression patterns change during neutrophil transmigration into the airspaces.

## Materials and methods

### Human LPS challenge and ARDS sample collection

Healthy subjects (age 25.8±5.5 years; mean±SD) were challenged with nebulised 0.9% saline or 50 μg LPS (*Escherichia coli* serotype O26:B6; Sigma, UK) in sterile saline, as part of a previously published study.^14^ BAL was performed 6 hours after challenge according to standard guidelines and prepared for analysis as previously described.^14^ Saline-challenged (n=5) and LPS-challenged (n=25) BAL fluid samples were used to measure the protein levels of CCL2 and CCL7 by ELISA (R&D Systems) according to the manufacturer's instructions.

Mechanically ventilated patients in the intensive care unit of the Royal Victoria Hospital, Belfast, Northern Ireland, were diagnosed with ARDS according to the consensus conference definition as previous described.^15^ Baseline BAL fluid samples, before administration of drug or placebo, from a randomised clinical trial (The HARP Study) were used for this study in order to measure CCL2 (n=18) and CCL7 (n=18) protein levels.^16^ Samples were prepared as previously described.^16^ A second cohort, again from two previously described, randomised, placebo-controlled clinical trials (the BALTI and VINDALOO trials),^17^ ^18^ were used to assess CCL2 and CCL7 protein levels in the BAL fluid from postoperative oesophagectomy patients who were at risk of developing ARDS, but did not, compared with those who did develop ARDS (n=20 for each, ARDS defined as PaO_2_:FIO_2_ ratio of<300 mm Hg). Random samples from both studies were used. Local institution and research ethics committee approval was obtained. Written informed consent from the legal representative of the patient or retrospective informed consent was obtained from the patient, if possible.

For flow cytometry analysis, adult patients over the age of 18 with suspected or confirmed community-acquired pneumonia with ARDS who required mechanical ventilation were recruited. ARDS was defined as meeting the American-European consensus definition of ARDS.^15^ Inclusion and exclusion criteria are defined in online supplementary table S1 and patient clinical details are described in online supplementary table S2. Ethics approval was obtained from the London-South East research ethics committee (ref: 13/LO/274). Informed consent was obtained from legal representatives of the subjects and retrospective informed consent from the individual where possible. Bronchoscopy was performed on mechanically ventilated patients via the endotracheal tube. BAL was performed in the lobe that appeared to be most affected on chest radiograph or CT. BAL was performed as previously described.^14^ Total cell counts were obtained using a haemocytometer.

10.1136/thoraxjnl-2016-208597.supp2 Supplementary data

### Neutrophil isolation

Neutrophils were isolated from the blood of healthy volunteers. About 20 mL blood was layered onto 10 mL 6% dextran (from leuconostoc spp, MR 45 000; Sigma-Aldrich) in sterile 0.9% saline and 20 mL phosphate-buffered saline (PBS). The solution was left to sediment for 45 min. The buffy coat layer was removed and centrifuged at 300 *g* for 5 min in 50 mL PBS, the leucocyte-rich layer was removed and resuspended in 55% Percol (GE Healthcare). A Percol gradient was prepared from 81% and 67% Percol and the resuspended leucocytes were layered on top (in 55% Percol). The Percol gradient was centrifuged for 30 min at 700 *g*. The neutrophil layer was removed and washed in PBS followed by centrifugation at 300 *g* for 10 min. Neutrophils were resuspended in 1 mL double-distilled H_2_O for 30 s and resuspended in 20 mL PBS. Cells were centrifuged and resuspended in RPMI-1640 (Sigma-Aldrich). Isolated neutrophils had a purity of >98% (see online supplementary figure S1).

### Neutrophil chemotaxis assay

ChemoTX plates (Neuro Probe) were used throughout (3 µm pores in a 96-well plate) employing 5×10^4^ neutrophils per well in RPMI-1640. Chemotaxis of isolated human neutrophils was measured in response to human BAL fluid (described above), with or without 10 µg/mL anti-human CCL2 or CCL7 neutralising antibody (anti-human CCL2 279-MC, R&D Systems, anti-human CCL7 AF-282-NA, R&D Systems), 10 µg/mL anti-human CXCL8 neutralising antibody (anti-human IL-8 AB-208-NA, R&D Systems) or 20 μg/mL polyclonal Ig control (R&D Systems) diluted 1:1 in RPMI-1640. The concentration of antibody used exceeded that required to neutralise the amount of chemokine in human ARDS BAL fluid (at least 10-fold excess; 10 μg/mL antibody has ND50 5–20 μg/mL chemokine). BAL fluid was incubated for 20 min with each neutralising antibody in the lower chamber, prior to the addition of 5×10^4^ neutrophils per well onto the upper membrane. At least 18 BAL fluid samples from different patients were used and each sample was run in triplicate. Neutrophils from the blood of multiple healthy human volunteers were used throughout. Recombinant human CXCL8 (IL-8), CCL2 and CCL7 (Peprotech) were used at various concentrations, were resuspended in RPMI-1640 and were placed in the lower chamber of the ChemoTX plate. Neutrophils were placed on the upper membrane and incubated at 37°C in 5% CO_2_ for 1 hour. Migrated neutrophils were counted using a haemocytometer and the chemotactic index was calculated (number of migrated neutrophils following treatment vs medium control).

### Flow cytometry analysis

About 30 mL of red cell lysis buffer (Roche, UK) was added to 3 mL of blood for 15 min. About 20 mL of PBS was added and sample was centrifuged at 300 *g* for 5 min. BAL fluid cell pellets were resuspended in 1 mL red cell lysis buffer for 10 min. Cells were resuspended in 1 mL PBS. Non-specific binding was blocked with 10% fetal bovine serum in PBS. Single-stained controls and fluorescence minus one controls were included. Neutrophils were gated using anti-CD14 (Becton Dickinson (BD)), anti-CD16 (R&D Systems) and anti-human leucocyte antigen-D-related (HLA-DR) (BD) antibodies (gating strategy is described in online supplementary figure S2). Chemokine receptor expression was assessed following incubation with anti- chemokine (C-X-C motif) receptor (CXCR)1 (BD), anti-CXCR2, anti-C-C chemokine receptor (CCR)1, anti-CCR2 and anti-CCR3 antibodies (all R&D Systems). Following 20 min incubation, samples were washed twice with 100 µL of 1% bovine serum albumin in PBS and centrifuged at 300 *g* for 3 min at each wash and the supernatant discarded. Cells were resuspended in 100 µL of 4% paraformaldehyde (PFA) and were kept at 4°C in the dark until acquisition on a FACS Verse flow cytometer (BD).

### Statistical analysis

Statistical analysis was performed using one-way analysis of variance with Newman-Keuls post-hoc test. p Values are presented within each figure.

## Results

### CCL2 and CCL7 are elevated in ARDS BAL fluid

**Figure 1:**
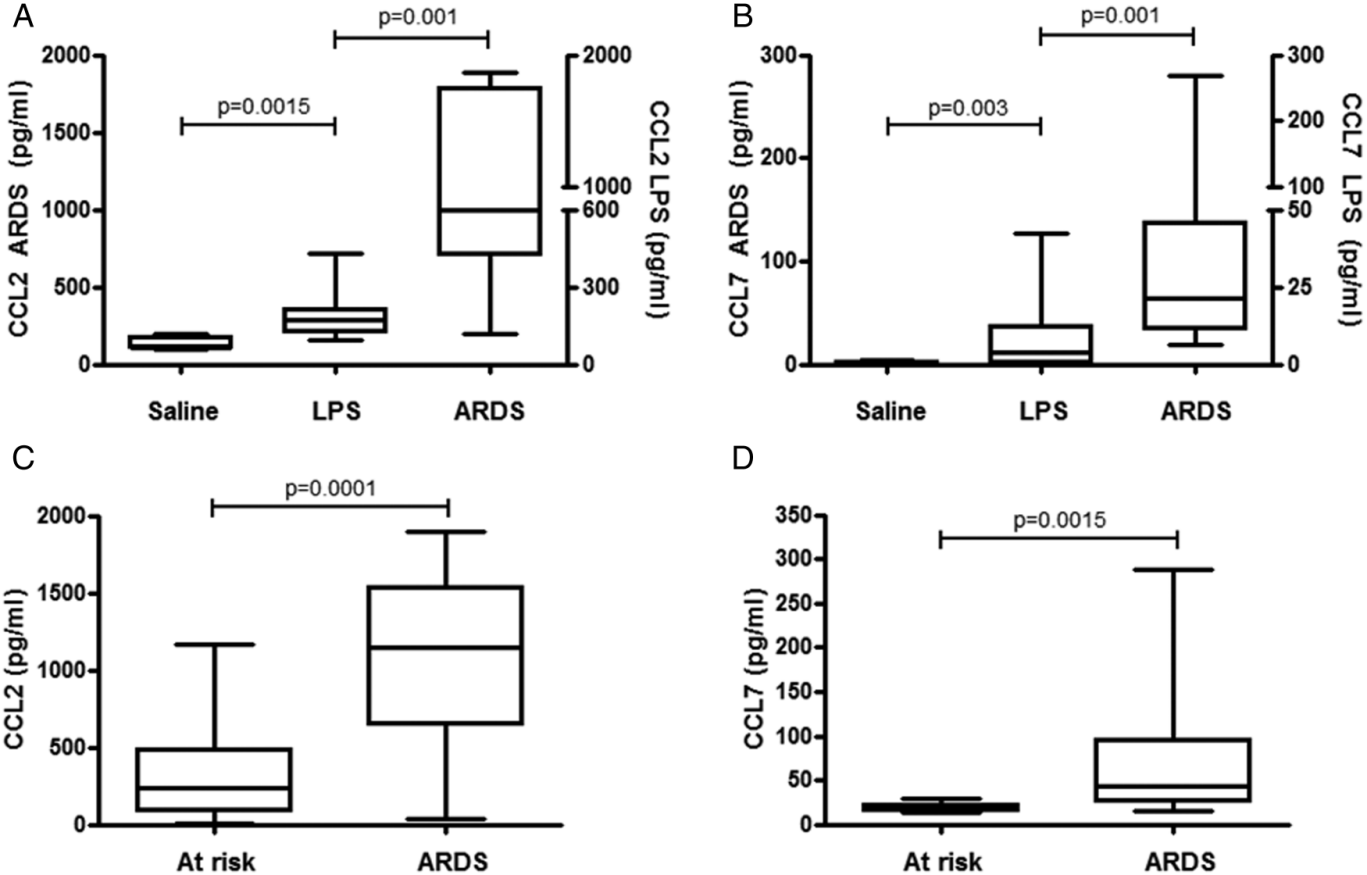
Plots showing th elevated bronchoalveolar lavage (BAL) fluid levels of chemokine (C-C motif) ligand (CCL)2 and CCL7 in a human model of acute lung inflammation and in patients with acute respiratory distress syndrome (ARDS). Healthy volunteers were challenged with 0.6% nebulised saline (n=5) or with 50 μg of nebulised lipopolysaccharide(LPS) (n=25) and BAL was performed 6 hours later. The levels of CCL2 (A) and CCL7 (B) were measured in BAL fluid recovered from saline and LPS-challenged volunteers by ELISA (right axis). BAL was also performed on patients with a clinical diagnosis of ARDS and the levels of CCL2 (A) and CCL7 (B) were measured (n=18) in recovered ARDS BAL fluid by ELISA (left axis). BAL fluid was also recovered from a second cohort of patients who underwent surgical oesophagectomy who were either at risk of developing ARDS (n=20) or who went on to develop ARDS (n=20). The levels of CCL2 (C) and CCL7 (D) were measured in recovered BAL fluid by ELISA. Power calculations were based on previous studies with α=0.05; power=0.8; difference between means=1.5 (50% reduction); SD=1.6. Statistical analysis was performed using one-way analysis of variance with Newman-Keuls post-hoc test.

We have previously reported that the chemokines CCL2 and CCL7 are elevated in the lungs of mice challenged with either LPS or infected with *S. pneumoniae* and that neutralising these chemokines inhibits neutrophil accumulation in BAL fluid.^12^ ^13^ In order to translate these findings into the human disease setting, we first collected BAL fluid from healthy volunteers who were challenged with LPS (50 mg) or saline. The levels of both CCL2 (figure 1A) and CCL7 (figure 1B) were increased in the BAL fluid isolated from LPS-challenged individuals. Furthermore, patients with a clinical diagnosis of ARDS had elevated levels of CCL2 (figure 1A) and CCL7 (figure 1B) in BAL fluid compared with normal controls and higher levels of these chemokines compared with LPS-challenged volunteers. In comparison, the levels of CXCL8 in ARDS BAL fluid previously reported by Craig *et al*^16^ (2011) ranged between 40 and 9721 pg/mL (mean 3277, SD 3684). In a second, independent cohort of patients with a clinical diagnosis of ARDS following oesophagectomy, levels of CCL2 (figure 1C) and CCL7 (figure 1D) were increased compared with postoperative patients at high risk of developing ARDS but remained free of clinical diagnosis. Collectively, these findings suggest that CCL2 and CCL7 may play a role in the pathogenesis of ARDS in humans.

### CCL2 and CCL7 significantly contribute to the chemotactic potency of ARDS BAL fluid

**Figure 2:**
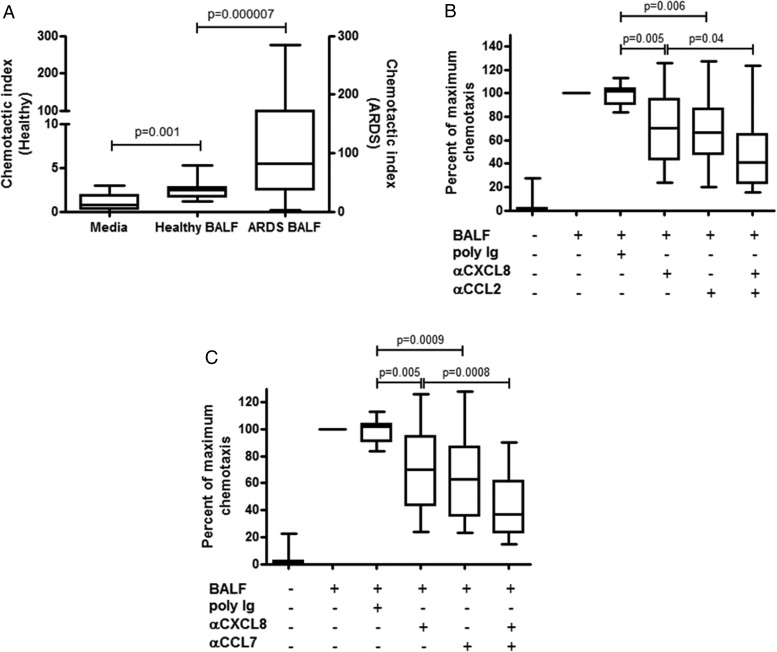
Chemokine (C-X-C motif) ligand 8 (CXCL8), chemokine (C-C motif) ligand (CCL)2 and CCL7 contribute to the neutrophil chemotactic activity of acute respiratory distress syndrome (ARDS) bronchoalveolar lavage fluid (BALF). Human neutrophils were isolated from the blood of healthy volunteers and chemotaxis was measured across 3 µm membranes (ChemoTX, NeuroProbe) in response to media alone, healthy volunteer BALF (n=11) or BALF from patients with ARDS (n=18) (A). Neutrophil chemotaxis was measured as the chemotactic index (number of migrated neutrophils following treatment vs medium control) after 1 hour (healthy BALF, left axis; ARDS BALF, right axis). Neutrophil chemotaxis towards BALF obtained from patients with ARDS was measured in the presence of polyclonal Ig control (20 μg/mL), neutralising anti- CXCL8) (10 μg/mL), anti-CCL2 (10 μg/mL) and a combination (10 μg/mL of each) of the two antibodies (B) or anti-CXCL8 (10 μg/mL), anti-CCL7 (10 μg/mL) and a combination (10 μg/mL of each) of the two antibodies (C). Antibodies were incubated in the presence of ARDS BALF for 20 min prior to the addition of isolated neutrophils. Differences in neutrophil chemotaxis between treatment groups were measured as the percentage of maximal chemotaxis to ARDS BALF alone (n=18 ARDS BALF samples using neutrophils isolated from multiple healthy human volunteers). Statistical analysis was performed using one-way analysis of variance with Newman-Keuls post-hoc test.

Having demonstrated that CCL2 and CCL7 are elevated in BAL fluid isolated from patients with ARDS, we next sought to elucidate whether CCL2 and CCL7 contribute to neutrophil recruitment in ARDS. First, we demonstrated that ARDS BAL fluid is highly chemotactic for human neutrophils compared with BAL fluid from healthy volunteers (figure 2A). In order to determine the contribution of CCL2 and CCL7 to the chemotactic activity of ARDS BAL fluid, we neutralised both chemokines using specific antibodies. Compared with the maximal chemotactic activity of ARDS BAL fluid, neutralising the classical neutrophil chemokine, CXCL8, significantly decreased neutrophil chemotaxis (figure 2B, C). Neutralising either CCL2 (figure 2B) or CCL7 (figure 2C) also decreased neutrophil chemotaxis towards ARDS BAL fluid, but a combination of either anti-CXCL8 plus anti-CCL2 (figure 2B) or a combination of anti-CXCL8 plus anti-CCL7 (figure 2C) caused a cumulative decrease in the neutrophil chemotactic response to ARDS BAL fluid. Taken together, these data suggest that CCL2 and CCL7 may contribute to the recruitment of neutrophils in ARDS by synergising with CXCL8.

### CCL2 and CCL7 enhance neutrophil chemotaxis to CXCL8

**Figure 3:**
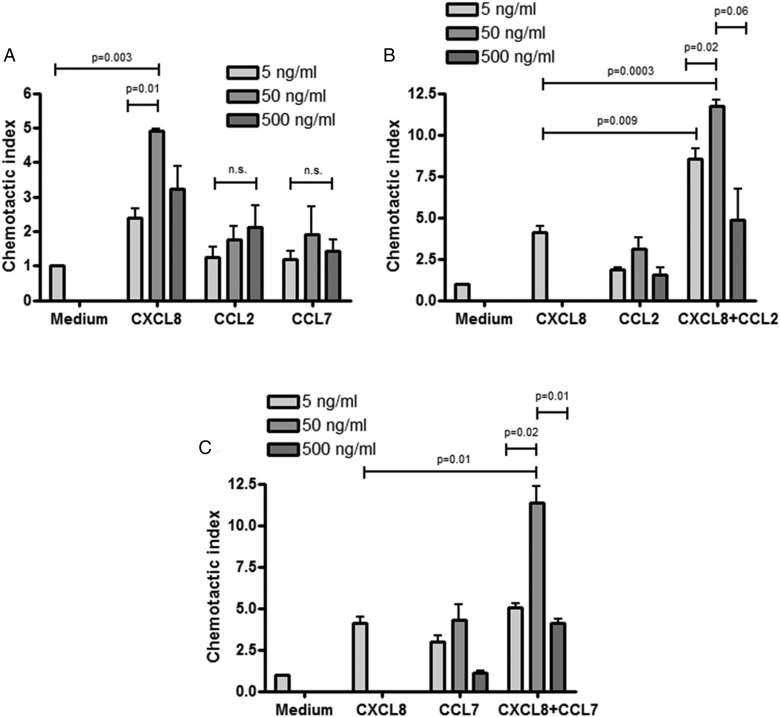
Human neutrophils migrate toward recombinant chemokine (C-X-C motif) ligand 8 (CXCL8), chemokine (C-C motif) ligand (CCL)2 and CCL7. Human neutrophils were isolated from the blood of healthy volunteers and chemotaxis was measured across 3 µm membranes (ChemoTX, NeuroProbe) in response to recombinant human CXCL8, CCL2 and CCL7 (A), over a range of concentrations as stated. Neutrophil chemotaxis was also measured in response to a suboptimal concentration of recombinant human CXCL8 (5 ng/mL) in combination with recombinant human CCL2 (B) or recombinant human CCL7 (C) across a range of concentrations as stated (n=3 independent experiments using different healthy volunteer donors). Neutrophil chemotaxis was measured as the chemotactic index (number of migrated neutrophils following treatment vs medium control) after 1 hour. Statistical analysis was performed using one-way analysis of variance with Newman-Keuls post-hoc test.

We next compared the neutrophil chemotactic activity of CCL2 and CCL7 with that of CXCL8. Neutrophils migrated towards CXCL8 in a concentration-dependent manner (figure 3A), with maximal chemoattraction at 50 ng/mL. CCL2 (figure 3B) and CCL7 (figure 3C) alone caused a small but non-significant amount of chemotaxis of neutrophils over a range of concentrations. However, when combined with a suboptimal dose of CXCL8 (5 ng/mL), both CCL2 (figure 3B) and CCL7 (figure 3C) significantly increased neutrophil chemotaxis. The data further suggest that CCL2 and CCL7 enhance neutrophil chemotaxis by synergising with CXCL8.

### BAL fluid neutrophils from patients with ARDS modify their chemokine receptor expression

**Figure 4:**
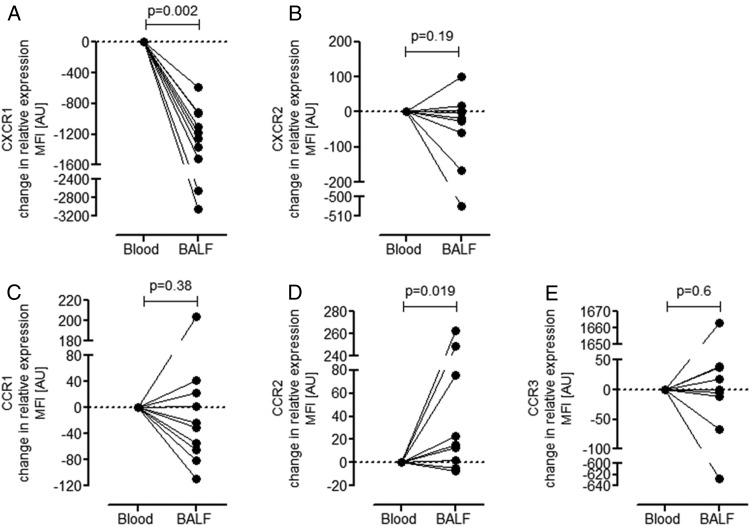
Neutrophils differentially regulate chemokine receptors during acute respiratory distress syndrome (ARDS). Bronchoalveolar lavage fluid (BALF) and blood was recovered from patients with a clinical diagnosis of ARDS (patient details in online supplementary table S2). Flow cytometry analysis of neutrophils was performed by gating on CD16+, CD14−, human leucocyte antigen-D-related (HLA-DR)− leucocyte populations. The neutrophil population was further verified by FSC and SSC properties (details in online supplementary figure S2). The expression of chemokine (C-X-C motif) receptor (CXCR)1 (A), CXCR2 (B), C-C chemokine receptor (CCR)1 (C), CCR2 (D) and CCR3 (E) on neutrophils from the blood of patients with ARDS was compared with neutrophils from the BALF of matching patients with ARDS (n=10). Data are represented as the ratio of mean fluorescent intensity (MFI) of each chemokine receptor expressed on BALF neutrophils versus blood neutrophils (direct measurements of MFI are presented in online supplementary figure S3). Statistical analysis was performed using a Wilcoxon signed rank test. FSC, forward scatter; SSC, side scatter.

We have previously demonstrated, in a mouse model of LPS-induced acute lung inflammation, that neutrophils can alter their expression of chemokine receptors when migrating into inflamed lung.^12^ Therefore, in order to determine whether the cell surface chemokine receptor expression on neutrophils changes during neutrophil transmigration in ARDS, we examined whether the surface expression of CXCR1, CXCR2 (receptors for CXCL8), and CCR1, CCR2 and CCR3 (receptors for CCL2 and CCL7) differed on neutrophils isolated from the blood and BAL fluid of patients with ARDS. The conventional neutrophil chemokine receptors CXCR1 and CXCR2 were highly expressed on neutrophils (CD16+, CD14−, HLA-DR−; gating strategy for BAL fluid and blood, see online supplementary figure S2) isolated from the blood of patients with ARDS (figure 4A). In contrast, neutrophils isolated from the ARDS BAL fluid of patient with ARDS exhibited a significant decrease in cell surface CXCR1 expression relative to the levels expressed by blood neutrophils (figure 4A). The expression of CXCR2 on ARDS BAL fluid neutrophils was more heterogeneous (figure 4B), with no significant alteration in expression levels. The expression of the CCL2 and CCL7 chemokine receptors CCR1, CCR2 and CCR3 on the blood neutrophils of patients with ARDS were low compared with that of CXCR1 and CXCR2 (figure 4C–E). The expression of CCR1 and CCR3 on ARDS BAL fluid neutrophils of patients with ARDS was not significantly different compared with the blood neutrophils of patients with ARDS (figure 4C, E). However, the expression of CCR2 was significantly elevated on neutrophils isolated from ARDS BAL fluid (figure 4D) relative to the expression levels measured on blood neutrophils (the mean fluorescent intensity for each chemokine receptor in the blood and BAL fluid is presented in online supplementary figure S3). Taken together, these data show that neutrophil chemokine receptor expression is plastic and changes during neutrophil transmigration from the blood to the airspaces during ARDS and these data further show that these neutrophils may gain responsiveness to CCL2 and CCL7 by expressing CCR2.

## Discussion

In the present study, we aimed to determine the role of CCL2 and CCL7 in influencing neutrophil chemotaxis in the context of ARDS. We have previously demonstrated that CCL2 and CCL7 are elevated in a mouse model of LPS-induced acute lung inflammation^12^ and a mouse model of *S. pneumoniae* infection.^13^ Furthermore, neutralisation of either CCL2 or CCL7 reduced the number of neutrophils isolated from the BAL fluid of LPS-treated mice, while neutralisation of CCL7 reduced the neutrophil numbers following pneumococcal infection. However, the contribution of these chemokines in the setting of human ARDS is not known.

ARDS is associated with an increase in several proinflammatory cytokines, chemokines and growth factors. Of note, IL-1β, tumour necrosis factor, IL-6 and CXCL8 are considered to be central mediators of the inflammatory immune response during ARDS pathogenesis.^19^ Activation of tissue-resident alveolar macrophages, alveolar epithelium and the endothelium establishes a lung environment conducive to the recruitment of inflammatory cells in the airspace.^6^ Neutrophils comprise the majority of cells recruited into the lung and although they are important for the clearance of pathogens, they are thought to contribute to the disruption of the epithelial-endothelial barrier in ARDS.^20^ The result of a damaged alveolar-endothelial unit is the leakage of protein-rich fluid into the airspace, which in turn compromises gaseous exchange and promotes respiratory failure.

The chemokine CXCL8 is considered to be the archetypal neutrophil chemoattractant and levels of CXCL8 have been directly associated with the number of neutrophils recruited into the inflamed lung during ARDS, as well as with disease severity and poor clinical outcome.^21–23^ Although CCL2 has previously been shown to be elevated in BAL fluid of patients with ARDS,^11^ its specific role in mediating neutrophil migration in this disease setting has not been comprehensively explored. Furthermore, the closely related chemokine family member, CCL7, has largely been overlooked in inflammatory lung diseases. CCL2 and CCL7 are conventionally considered to mediate the recruitment of monocytes and macrophages into sites of inflammation in response to various inflammatory insults,^24^ including LPS,^25^ and the bacterium *Listeria monocytogenes*.^26^ In humans, CCL2 and CCL7 have been shown to differentially induce the chemotaxis of macrophages, with CCL7 being the only chemokine to induce the migration of M1 and M2 macrophages.^27^ More recently, it has been demonstrated that CCL2 and CCL7 can also function as chemoattractants for mouse^28^ ^29^ and human neutrophils.^30^ We now show, for the first time, that CCL2 and CCL7 contribute to the neutrophil chemotactic activity of ARDS BAL fluid and that they synergise with CXCL8 to promote neutrophil chemotaxis.

Even though they are closely related chemokines, CCL2 and CCL7 differ in their biophysical characteristics. While CCL2 is a ligand restricted to the CCR2 receptor,^31^ CCL7 is more promiscuous and can bind and signal via CCR1, CCR2 and CCR3^32^ and may therefore be able to influence the migratory capacity of multiple cell types compared with CCL2. In addition, CCL7 is able to bind to glycosaminoglycan (GAG) polysaccharides with higher affinity, so that CCL7 may form a more robust chemotactic gradient compared with CCL2.^33^ This additional GAG-binding property of CCL7 may also account for lower amounts of detectable unbound CCL7 in body fluids compared with CCL2. These are important considerations which need to be taken into account when designing new therapeutics that target chemokine family members.

In both chemokine neutralisation studies and in chemotaxis assays using recombinant human chemokines, we demonstrated that a level of synergy exists between CXCL8 and CCL2 or CCL7 in promoting neutrophil migration. It has previously been shown that CCL7 can synergise with CXCL6, in a mouse model of peritoneal inflammation^34^ and with CXCL10 in mouse models of acid-induced lung injury and influenza infection.^29^ Certain chemokines are also able to act cooperatively by forming heterodimers. For example, CCL5 and CXCL4 heterodimers are elevated in ARDS and in mouse models of lung injury, while destabilising these complexes reduced sepsis and LPS-induced lung injury in mice.^35^ Although CXCL8, and most other chemokines, can form homodimers and even tetramers, the different three-dimensional configuration of CXC-chemokines compared with CC-chemokines makes heterodimer formation between family members more likely than mixed CXC-CC heterodimers.^36^ For example, CXCL8 readily forms a heterodimer with CXCL4 (platelet factor 4), while CCL2 can form heterodimers with CCL5 and CCL8. However, there is currently no direct evidence that CXCL8 can form heterodimers with either CCL2 or CCL7. In addition, CCL7 is considered to function as an obligate monomer and therefore highly unlikely to dimerise under these conditions.^33^ Furthermore, dimerisation is largely thought to increase the stability of chemokines in physiological fluids but is independent of receptor activation.^37^

The synergistic effect between CXCL8 and CCL2 or CCL7 is therefore most likely due to increased receptor occupancy on neutrophils, which in turn accounts for heightened chemotaxis towards multiple chemokines. Previous studies using recombinant chemokines reported that both CCL2 and CCL7 can synergise with CXCL8 to enhance neutrophil migration, with CCL7 being more effective than CCL2.^38^ ^39^ Our data confirm the synergistic effect between these chemokines and highlight its potential importance in the ARDS disease setting. Human neutrophils isolated from the BAL fluid of patients with COPD have also been reported to express higher levels of CCR1, CCR2 and CCR3 and CCL2, CCL3, CCL4 and CCL11 were able to induce chemotaxis of neutrophils isolated from BAL fluid of patients with COPD.^30^ This and our current study therefore suggest that neutrophils are capable of responding to a number of CC and CXC chemokines other than CXCL8 and its closely related family members.

We previously demonstrated that in mouse models of acute lung injury, neutrophils differentially express CXCR and CCR chemokine receptors on their cell surface depending on the tissue micro-compartment in which they reside.^12^ In order to determine if this is also the case in human ARDS, we analysed neutrophils from the blood and BAL fluid of patients with ARDS. Compared with blood neutrophils, BAL fluid neutrophils expressed significantly lower levels of CXCR1 but higher levels of CCR2, suggesting that human neutrophils can also gain responsiveness to different chemokine subsets depending on the tissue microenvironment in which they reside. The increased expression of CCR2 on ARDS BAL fluid neutrophils further suggests that neutrophils are capable of responding to CCL2 and CCL7 within airspaces in this disease setting, although the factors that regulate CCR2 expression on neutrophils will need to be fully explored in the future.

Our study has some limitations in that although we demonstrated that CCL2 and CCL7 levels are elevated in ARDS, we would have ideally also included studies using a comparator control group of mechanically ventilated patients without ARDS. To address this, we analysed a separate cohort of oesophagectomy patients with and without ARDS and demonstrated that CCL2 and CCL7 levels are significantly increased in patients who went on to develop ARDS.

In conclusion, our data indicate that CCL2 and CCL7 levels are elevated in BAL fluid samples from patients with ARDS and that these chemokines contribute to the chemotactic activity of ARDS BAL fluid by synergising with the conventional neutrophil chemoattractant, CXCL8. Furthermore, neutrophils differentially regulate the expression of CXCR1 and CCR2 when migrating into the bronchoalveolar compartment, thereby enabling them to gain responsiveness to different chemokine subsets depending on the tissue microenvironment in which they reside. Our neutrophil receptor expression studies further highlight that there is patient heterogeneity in terms of expression patterns. A clearer understanding of patient heterogeneity in ARDS is critical for the successful design and implementation of both new therapeutics and clinical trials in this disease setting.
