# Supplementary material for: Evidence for chemokine synergy during neutrophil migration in ARDS
Source: Thorax. 2016 Aug 5;72(1):66–73. doi: 10.1136/thoraxjnl-2016-208597 (PMC5329051; doi:10.1136/thoraxjnl-2016-208597)
Supplement: Supplementary data [file thoraxjnl-2016-208597supp.pdf]

Supplementary Table 1.

Inclusion Criteria	*Exclusion Criteria*
All adults above 18 years of age	Pregnant and those under the age of 18 years
Suspected or confirmed CAP	Those with clinical signs of left ventricular failure, or
Defined ARDS on mechanical ventilation	Pulmonary capillary wedge pressure greater than 18 mmHg, or
New infiltrate on chest radiograph, with one or more:	Evidence suggesting a cardiac basis for the pulmonary oedema
Cough	Patients on non-invasive mechanical ventilation
Sputum production	Patients on anticoagulants except those on prophylactic dose low molecular weight heparin
Dyspnea	Patients with active bleeding
Core body temperature >38.0°C	Patients with active cancer (refers to malignancy which is recently diagnosed, under treatment, or relapsed)
Auscultatory findings of abnormal breath sounds and rates	Patients that received solid-organ transplant or bone-marrow transplant within the last year
Leukocyte count >10 or <4 x 109 cells/L	Mechanically or chemically-induced ALI/ARDS (including burns, trauma, and near drowning).
PaO2/FiO2) ratio of less than 300 mm Hg	Patients with end-stage chronic lung disease
Pulmonary wedge pressure of less than 18 mm Hg	Patients that had any one of the following:
Absence of clinical evidence of left atrial hypertension	Platelet count <50,000/mm3
	INR >1.5
	Significant potential for DIC - Prolonged INR and low fibrinogen
	Those who had received clotting factor replacement in the last 1 week.
	Patients with bleeding disorders within the past 6 weeks or vasculitis with diffused alveolar haemorrhage
	Patients having received warfarin within 7 days

Supplementary Table 1. Exclusion and Inclusion criteria.
Inclusion and exclusion criteria for patients recruited to the ICU at UCLH under the London - South East research ethics committee (ref: 13/LO/274).
CAP - community acquired pneumonia
INR - international normalized ratio
DIC - disseminated intravascular coagulation

Supplementary Table 2.

	CAP-ARDS (n=12) Count/Median (IQR)
Age (years)	76 (68, 83)
Gender (M)	8
ICU Mortality	42%
Day of ICU Admission	
APACHE II score	37 (32, 43)
SOFA score	10 (8, 12)
P:F ration (Kpa)	25 (21, 27)
Neutrophils ($\times 10^9$/L)	8 (6, 9)
Platelets ($\times 10^9$/L)	178 (114, 234)
CRP (mg/L, <5)	227 (114, 234)
ALT (7 – 56)	39 (127, 152)
INR (<1.5)	1 (1.1, 1.2)
PT (<13 s)	13 (13, 15)
APTT (30 – 40 s)	48 (44, 61)
Inotropes (n)	7
During ICU stay	
Inotropes (n)	11
RRT (n)	4

Supplementary Table 2. Patient clinical details.

Supplementary figure 1.

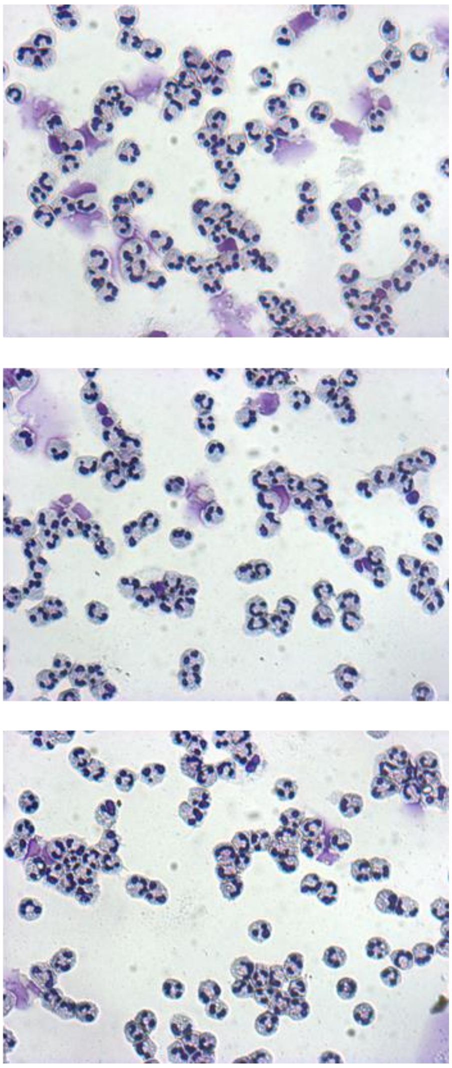

Supplementary Figure 1. Neutrophil isolation from healthy volunteers. Neutrophils were isolated from the blood of healthy volunteers using dextran sedimentation and percol gradient centrifugation, as described in the Methods section. The purity of neutrophils isolated by this method was assessed by cytospin, following staining with Rapid Romanowski Stain (TSC Biosciences) and assessed under light microscopy. Neutrophils were routinely isolated with a purity of 98% or above (>98%). Three micrographs from separate isolations are presented (A, B and C).

Supplementary figure 2.

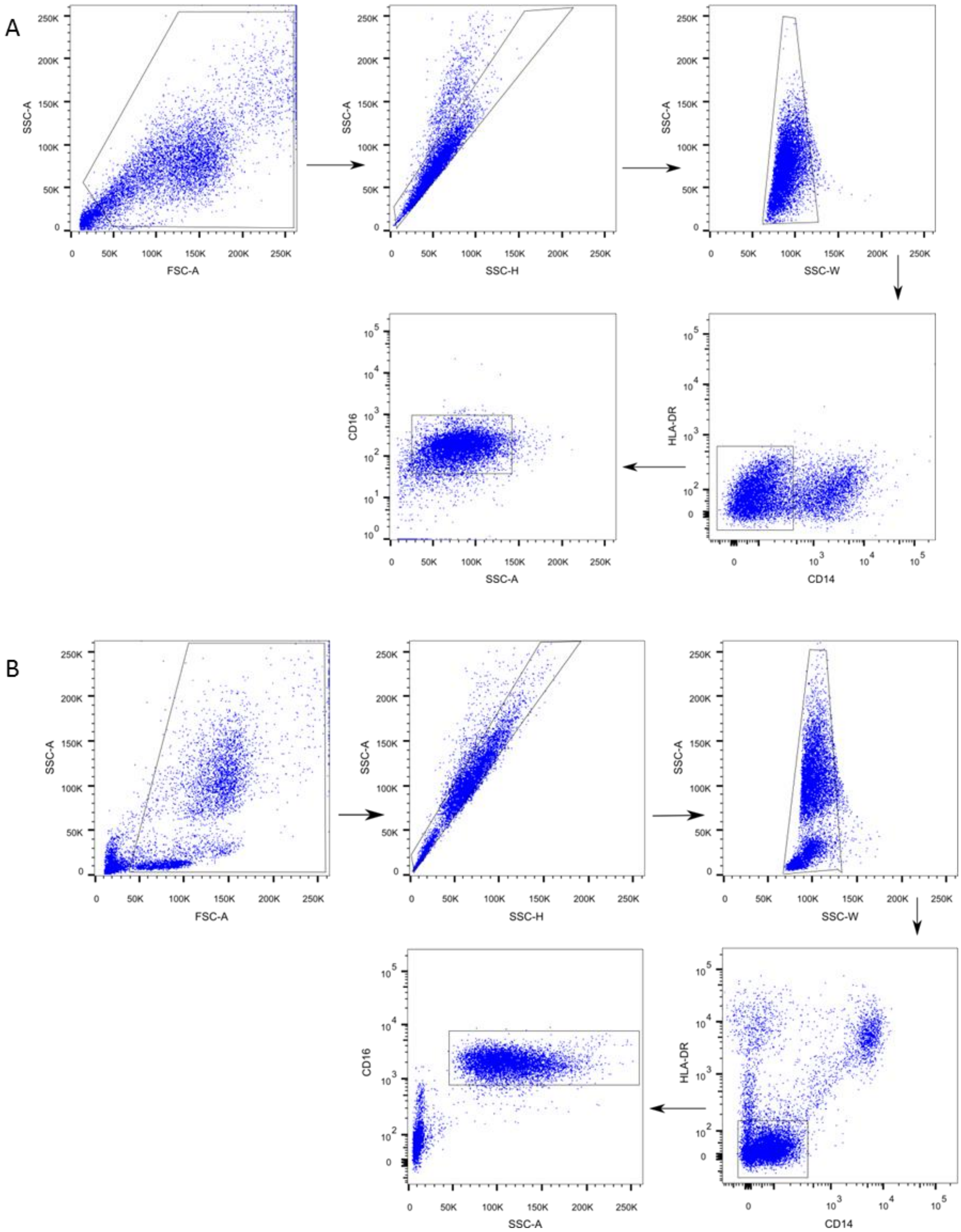

Supplementary Figure 2. Gating strategy used on neutrophils isolated from the BAL fluid and blood of ARDS patients. Following red blood cell lysis (30 sec in distilled H2O), neutrophils were incubated with the antibody cocktail described in the Methods section. FSC vs SSC was used to identify the leukocyte population and exclude thrombocytes and cell debris. SSC-A vs SSC-H and SSC-A vs SSC-W was used to exclude cell doublets. CD14+, HLA-DR+ cells were then excluded to remove monocytes, macrophages and dendritic cells from the analysis. Neutrophils were finally gated as CD16+ (CD14-, HLA-DR-). The chemokine receptor expression (CXCR1, CXCR2, CCR1, CCR2 and CCR3) was then analysed on the neutrophil population. (A) Represents BAL fluid neutrophils and (B) blood neutrophils.

Supplementary figure 3.

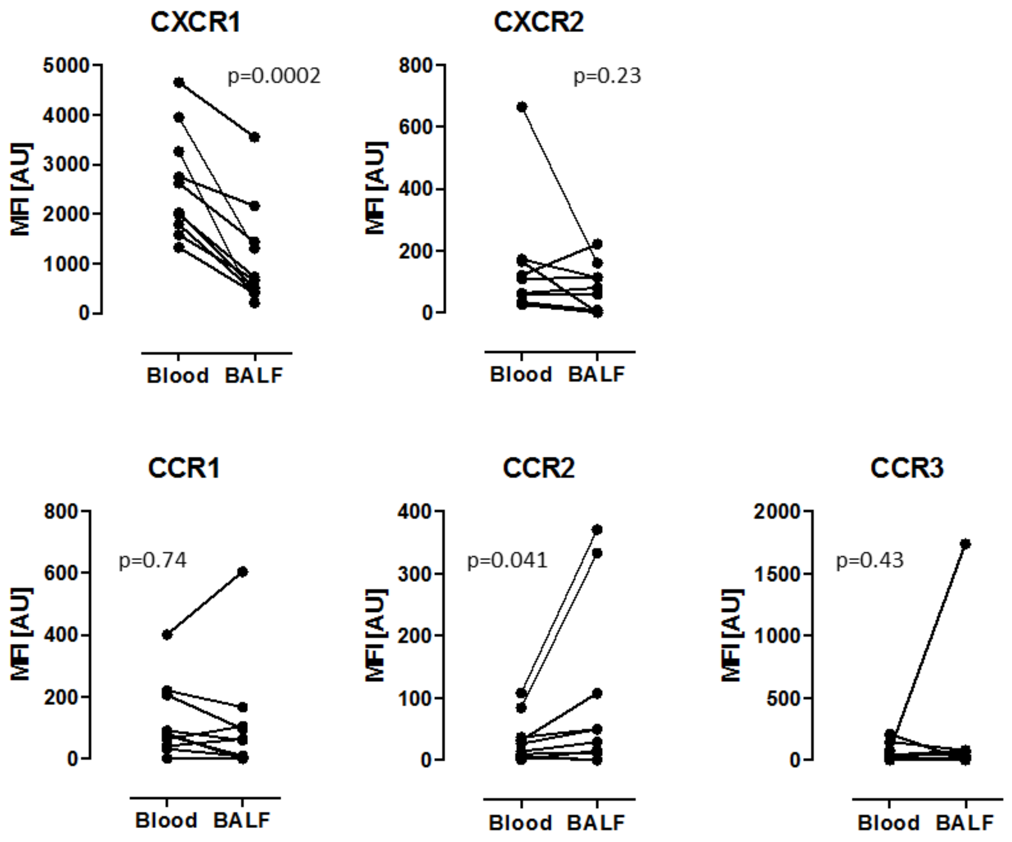

Supplementary Figure 3. Mean fluorescent intensity (MFI) of blood and BAL fluid neutrophils from ARDS patients. Data is presented as the actual MFI for each chemokine receptor expressed on the surface of neutrophils isolated from the blood or BAL fluid of ARDS patients. MFI was normalised to fluorescence minus one controls and negative values adjusted to zero. A paired t-test was performed on each chemokine receptor analysed. Only CXCR1 and CCR2 showed significant differences between blood and BAL fluid neutrophils.
